# Application of Radiofrequency Ablation for the Treatment of Cutaneous Hemangioma and Vascular Malformation in a Cockscomb Model

**DOI:** 10.1155/drp/2012304

**Published:** 2025-05-25

**Authors:** Hong-long Chen, Dong-mei Li, Xi-sheng Lin, Xiao Zhang, Tao Chen, Wei Chen, Yue-ming Gao

**Affiliations:** ^1^Department of Rehabilitation Medicine, The Second Medical Center and National Clinical Research Center for Geriatric Diseases, Chinese PLA General Hospital, Beijing, China; ^2^Department of Neurology, The Second Medical Center and National Clinical Research Center for Geriatric Diseases, Chinese PLA General Hospital, Beijing, China; ^3^Ninth Departments of Health Care, The Second Medical Center and National Clinical Research Center for Geriatric Diseases, Chinese PLA General Hospital, Beijing, China

**Keywords:** bleomycin, hemangioma, radiofrequency ablation, vascular malformation

## Abstract

**Background:** Radiofrequency ablation (RFA) is an emerging technology for the effective treatment of cutaneous hemangioma and vascular malformation. However, there are few histopathological studies on the treatment of this disease with RFA.

**Objective:** This study aimed to investigate the effect of RFA and associated histopathological changes in a cockscomb model of cutaneous hemangioma and vascular malformation.

**Methods:** Thirty-two Leghorn chickens were randomly divided into two groups: RFA group (treated with RFA; 220 V, pulse rate: 15 ms) and control group (treated with 1 mg/mL bleomycin). At 3, 7, 14, and 28 days after treatment, histopathological changes in the cockscomb tissues were observed visually and microscopically using hematoxylin and eosin staining and Masson's trichrome staining. The rates of capillary reduction and collagen proliferation were examined.

**Results:** The cockscomb in the RFA group developed scabs earlier than that in the bleomycin group, and the scabs were darker and more clearly defined. The RFA group showed a more severe inflammatory reaction than the bleomycin group. At 28 days, most scabs had fallen off in both groups, and the boundary was clearer in the RFA group. At 3, 7, and 14 days, the number of capillaries decreased in both groups, with a more obvious decrease in the RFA group. From Days 3 to 28, the number of capillaries in the RFA group showed a trend of gradual increase, whereas that in the bleomycin group showed a trend of gradual decrease, but there was no significant difference between the two groups at 28 days (*p* > 0.05). The collagenous fibers of cockscomb showed a trend of gradual increase in both groups. The collagenous fiber hyperplasia was higher in the RFA group than in the bleomycin group at 14 and 28 days (*p* < 0.01).

**Conclusion:** RFA significantly reduced the capillary number and promoted tissue fibrosis. Compared with bleomycin, RFA showed a better effect and with no obvious side effects in treating a cockscomb model of cutaneous hemangioma and vascular malformation.

## 1. Introduction

Vascular abnormalities are a group of lesions with heterogeneous clinical manifestations, anatomical involvement, and treatment response. In 1996, the International Society for the Study of Vascular Anomalies adopted Mulliken's nomenclature of congenital vascular lesions [1]. This nomenclature divides the disease into two categories according to cell characteristics and biological behavior-vascular tumors and vascular malformations. Vascular tumors may subside as the patient's age increases, but vascular malformations will increase and will never subside on their own. Vascular abnormalities in visible parts of the body may lead to serious psychological and social problems, which have a complex impact on patients and their families. Most skin vascular tumors and vascular malformations require treatment, and early active intervention is recommended. For hemangiomas of nonsurface esthetic sites with limited range and no complications, the treatment strategy of observation and waiting can be selected [2, 3].

Although recommendations and guidelines have been developed and updated, interventional radiologists, physicians, and surgeons involved in the treatment of such conditions still have problems in diagnosis and treatment [4]. There are a variety of treatments for vascular malformations, including oral drugs, sclerotherapy, embolization, electroacupuncture therapy, radiofrequency ablation (RFA), and laser and surgical resection. So far, there is no reliable data for the treatment of vascular malformations in large samples [5–7]. The challenges of treatment include avoiding devastating damage and dysfunction to patients when removing abnormal vascular tissues. Seeking safe, effective, and minimally invasive treatment methods has always been the focus of interventional radiologists, physicians, and surgeons [8, 9]. However, comparative studies on the effects of different treatment methods on angiogenesis and collagen fibers are still limited, which makes clinicians face difficulties in selecting treatment options. A variety of treatments including bleomycin have been discussed in the existing literature, but their side effects and efficacy are quite different in different patients [10, 11]. RFA, as an emerging treatment method, has showed certain effectiveness and safety in the treatment of other types of tumors, including the treatment of hemangiomas [12, 13]. However, there is still a lack of systematic comparative study on the application of these two treatments in cockscomb hemangioma and their effects on angiogenesis and collagen fibers.

RFA is a minimally invasive, safe, and effective treatment technique. It is widely used in the treatment of solid tumors, varicose veins, and vascular abnormalities (such as hemangiomas, port wine stains, venous malformations, and lymphatic malformations), especially in areas with high appearance and function requirements [12, 14, 15]. Although the treatment principles and equipment parameters of different RFA instruments are not completely consistent, the treatment methods are basically to destroy the hemangioma and malformed vascular tissue through the heat generated by radiofrequency radiation, resulting in tissue necrosis, atrophy, and absorption [16]. However, RFA therapy for vascular abnormalities is mostly clinical treatment, and there are few basic studies on the use of RFA for the treatment of this disease.

In this study, we focused on the cockscomb (cockscomb is a mature experimental model of hemangioma and vascular malformation), especially the growth of adolescent cockscomb, which has the dual characteristics of hemangioma and vascular malformation [17, 18]. The study aims at angiogenesis and collagen fiber hyperplasia in the model of cockscomb hemangioma. Although previous studies have achieved a preliminary understanding of the mechanism of hemangioma formation in this field, the evaluation of the efficacy of different treatment methods still needs to be further studied [17–19]. By comparing the effects of bleomycin and RFA on angiogenesis, collagen fiber hyperplasia, and tissue healing, this study aims to evaluate the effectiveness of the two treatment methods from the perspective of histopathology and provide a scientific basis for future treatment options. The ultimate goal of this study is to provide a reference for clinicians in the selection of treatment options and to promote the progress of hemangioma treatment.

## 2. Materials and Methods

### 2.1. Experimental Animals

The chicken comb has been described as a well-established model for hemangioma and vascular malformation [17, 18]. In this study, 32 male Leghorn chickens aged 8–12 weeks were provided by the Animal Experiment Center of the General Hospital of the People's Liberation Army. All animals were housed in the same laboratory under clean conditions with free access to water. The experimental procedures complied with the “Regulations on the Administration of Laboratory Animals” of the People's Republic of China and ethical requirements. The 32 Leghorn chickens were randomly divided into two groups: the bleomycin group and the RFA group. In the bleomycin group, three points were selected as injection sites on each chicken comb, with 1 mg/mL bleomycin administered per site. In the RFA group, 4-5 circular areas were selected as RFA zones on each chicken comb. At 3, 7, 14, and 28 days postexperiment, eight chickens were randomly selected for comb observation and photography.

### 2.2. RFA Protocol

After intramuscular injection of xylazine (0.1 mg/kg) for anesthesia in experimental chickens, the bleomycin group was marked with three points on the comb using gentian violet. Each marked point received an intratissue injection of 1 mL bleomycin solution (1 mg/mL concentration). For the RFA group, 4-5 circular areas (1 cm diameter) were marked with gentian violet on the comb as ablation zones. The RFA device was adjusted to 220 V and 15 ms pulse current frequency. The treatment electrode was inserted approximately 2-3 mm into the comb surface tissue within each designated ablation zone, with each ablation lasting 1-2 s. Sequential ablation was performed across all marked areas with 4 mm spacing between electrode insertions until the entire treated comb surface turned white (treatment endpoint). Considering the comb thickness of 8–10 mm, interventions (bleomycin injection or RFA) were performed unilaterally on one side of the comb, while the untreated contralateral side served as self-control. Postprocedure chickens were maintained under standardized housing conditions with free access to food and water until tissue collection.

### 2.3. General Observation

The general condition of the comb, including shape, color, thickness, and surface characteristics, was observed and photographed before the experiment. After the experiment, observations were made regarding comb color changes, skin white spot, edema, surface tissue necrosis, scabs, scab shedding, and shedding time distribution. At 3, 7, 14, and 28 days after the experiment, eight randomly selected chickens were assessed and photographed, the experimental animals were anesthetized, and the tissues were cut and examined using light microscopy.

### 2.4. Histology

At 3, 7, 14, and 28 days after the experiment, biopsy specimens were fixed in 10% buffered formalin, dehydrated in graded ethanol, cleared in xylene, embedded in paraffin, cut into 3 mm thick sections, and stained with hematoxylin and eosin (H&E). Five high-power visual fields (400×) were randomly selected for each section to calculate the average number of capillaries. The rate of capillary reduction in the cockscomb was calculated according to the following formula: (average number of capillaries on the experimental surface−average number of capillaries on the control surface)/average number of capillaries on the control surface × 100%.

At 3, 7, 14 and 28 days after the experiment, biopsy specimens were fixed in 10% buffered formalin, dehydrated in graded ethanol, cleared in xylene, embedded in paraffin, cut into 3 mm thick sections, and stained with Masson's trichrome (Masson). With Masson's trichrome, connective tissue and collagen fibers are stained green, smooth muscle is stained red, and the nucleus is stained brown. Tissue section images were collected: The light lens was lowered to high magnification (400×), the lighting intensity was adjusted to the same level, and five randomly selected nonrepetitive visual fields for each Masson-stained tissue specimen were imaged successively. Subsequently, the same researcher processed the images using the color range function of Photoshop software to calculate the pixel value of the green collagen fiber area in each image. The average pixel value of the collagen fiber area of each section was estimated, and the collagen fiber proliferation rate in the comb was calculated according to the following formula: (average pixel value of collagen fiber area on the experimental surface−average pixel value of collagen fiber area on the control surface)/total pixel value of each image × 100%.

### 2.5. Statistical Analysis

Statistical analysis was conducted with SPSS 17.0. Data were expressed as mean ± standard deviation (SD). One-way analysis of variance test was used to compare the mean number of capillaries and the amount of collagen fiber expression between the two groups. Differences were considered statistically significant at *p* < 0.05.

## 3. Results

### 3.1. Clinical Response

**Figure 1:**
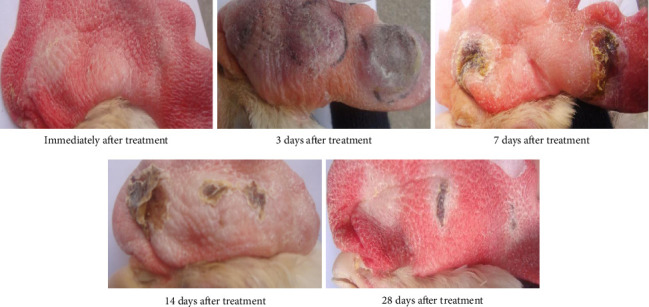
Macroscopic changes in cockscomb after bleomycin injection.

In the bleomycin (control) group, immediately after injection, a white skin lesion formed at the injection site. At 3 days posttreatment, significant swelling appeared at the injection site with purplish-red discoloration in the treated area. By Day 7, obvious scabbing was observed at the treatment site, surrounded by pale yellow coloration with persistent notable swelling. At Day 14, visible scabbing remained though swelling showed partial reduction compared to Day 7. By Day 28, the injection site exhibited pale grayish discoloration with residual scabbing but no apparent edema (Figure 1).

**Figure 2:**
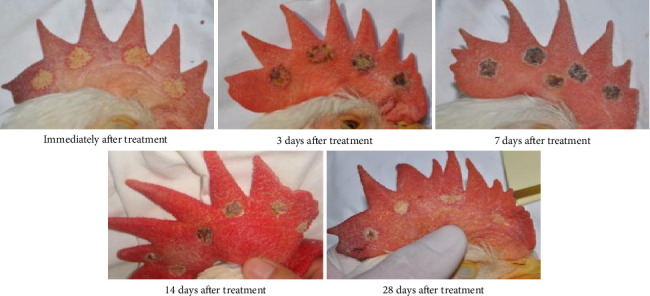
Macroscopic changes in cockscomb after radiofrequency ablation.

In the RFA group, immediately after treatment, the surgical site displayed well-defined circular yellowish-white lesions. At 3 days postoperation, black eschar began forming around the treatment area with mild swelling that was significantly milder compared to the bleomycin group. By Day 7, swelling had largely subsided, and the treatment area showed distinct circular black eschar formation, contrasting with the bleomycin group where swelling remained prominent. At Day 14, partial eschar shedding occurred with complete resolution of swelling, unlike the bleomycin group. By Day 28, most eschar had shed, leaving a grayish-white surgical area (Figure 2), demonstrating comparable efficacy to the bleomycin group.

### 3.2. Histology

**Figure 3:**
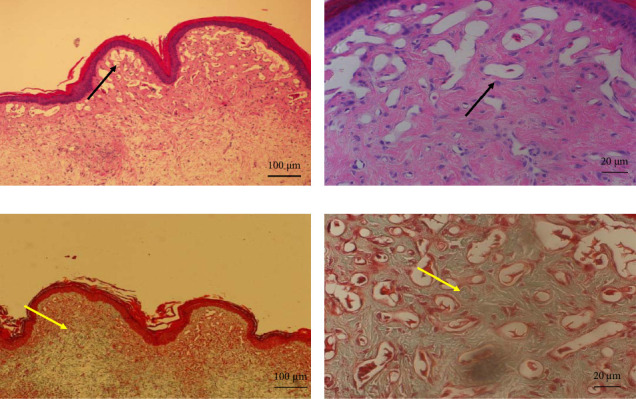
Photomicrographs of normal cockscomb: (a) hematoxylin and eosin (H&E) staining, 100× magnification; (b) H&E staining, 400× magnification; (c) Masson's trichrome staining, 100× magnification; (d) Masson's trichrome staining, 400× magnification. The black arrow represents normal capillaries in the comb. The yellow arrow represents the collagen fibers of cockscomb tissue.

**Figure 4:**
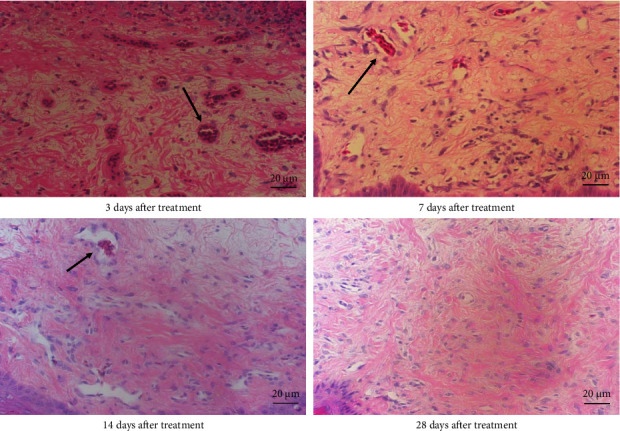
Hematoxylin and eosin staining of cockscomb tissue in the bleomycin group at 3, 7, 14, and 28 days after treatment (400× magnification). The black arrow represents normal capillaries in the comb.

**Figure 5:**
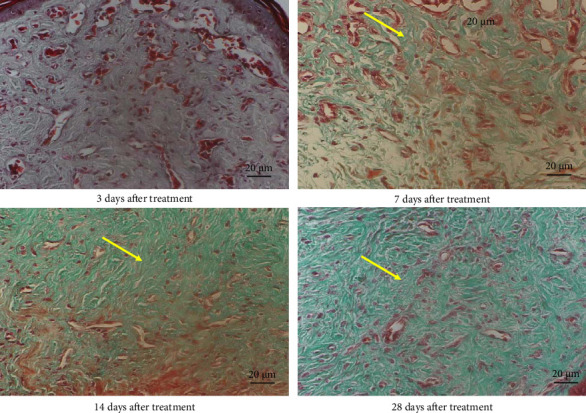
Masson's trichrome staining of cockscomb tissue in the bleomycin group at 3, 7, 14, and 28 days after treatment (400× magnification). The yellow arrow represents the collagen fibers of cockscomb tissue.

Normal chicken comb tissue exhibited neatly arranged epithelial cells, with abundant subcutaneous capillaries displaying regular morphology. Vascular spaces contained adipose tissue and loose connective tissue (including some collagen fibers) (Figure 3). Following bleomycin injection, the bleomycin group showed gradual reduction in capillary numbers and progressive collagen fiber deposition. Early-stage changes included inflammatory cell infiltration and interstitial edema at 3 days postinjection. Subcutaneous capillaries demonstrated destructive fusion with perivascular inflammatory cell infiltration, marked interstitial edema, and minimal collagen fiber proliferation. By Days 7 and 14, capillary numbers further decreased while collagen fiber deposition increased substantially. At 28 days, capillaries were significantly reduced and tissues became densely packed with collagen fibers (Figures 4 and 5). After RFA, tissues at 3 days posttreatment revealed thickened epidermal basement membrane, markedly diminished capillaries, prominent inflammation/edema, and sparse collagen deposition. By Day 7, basement membrane thickness reduced with slight capillary recovery, attenuated inflammation/edema, and evident collagen proliferation.

**Figure 6:**
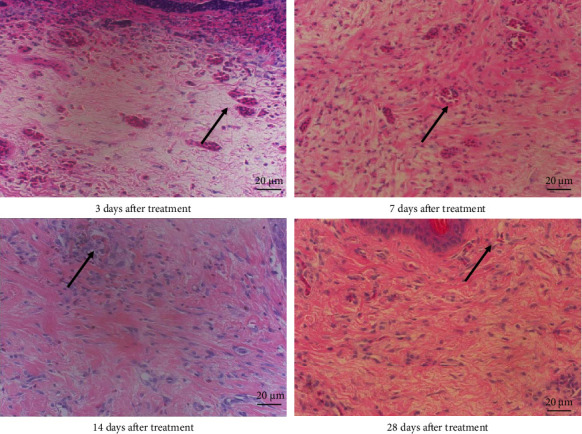
Hematoxylin and eosin staining of cockscomb tissue in the radiofrequency ablation group at 3, 7, 14, and 28 days after treatment (400× magnification). The black arrow represents normal capillaries in the comb.

**Figure 7:**
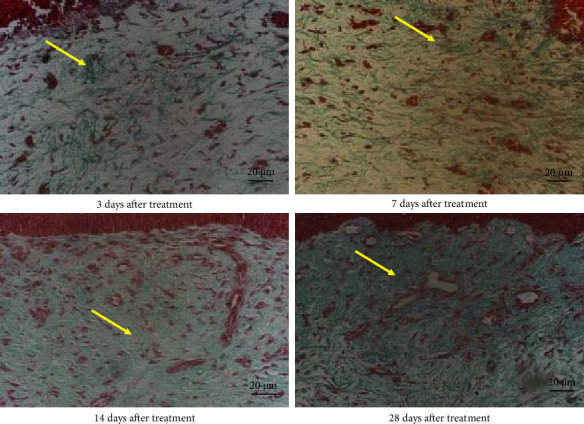
Masson's trichrome staining of cockscomb tissue in the radiofrequency ablation group at 3, 7, 14, and 28 days after treatment (400× magnification). The yellow arrow represents the collagen fibers of cockscomb tissue.

At 14 days, epithelial cells regained relative regularity, capillaries showed further regeneration, inflammation/edema nearly resolved, and collagen deposition remained pronounced. No significant capillary changes were observed with persistent collagen deposition at 28 days post-RFA (Figures 6 and 7).

### 3.3. Capillary Number

**Figure 8:**
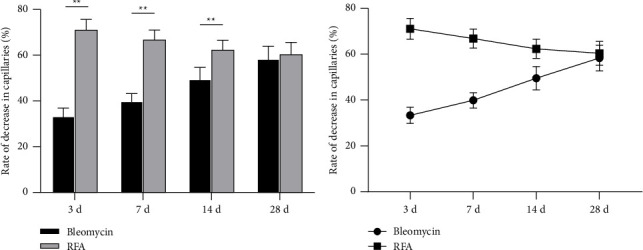
(a) Comparison of the rate of decrease in capillaries (%) between the bleomycin group and radiofrequency ablation (RFA) group on Days 3, 7, 14, and 28 after treatment; (b) trends of capillary changes on Days 3, 7, 14, and 28 after treatment. ^∗∗^Compared with the bleomycin group, *p* < 0.01.

This study observed changes in the number of crown capillaries in chickens at 3, 7, 14, and 28 days posttreatment. The bleomycin group showed a gradual decreasing trend in the capillary number over time, with the capillary reduction rate exhibiting a gradually increasing trend. Following RFA, the reduction in capillary numbers displayed a gradual declining trend, and the capillary reduction rate progressively decreased, though the temporal effect of RFA on crown capillary numbers was not statistically significant. Significant differences in capillary counts were observed between the bleomycin and RFA groups at 3, 7, and 14 days posttreatment (*p* < 0.05), but no significant intergroup difference was detected at 28 days (*F* = 0.025, *p* > 0.05; Table 1, Figure 8).

### 3.4. Collagen Hyperplasia

**Figure 9:**
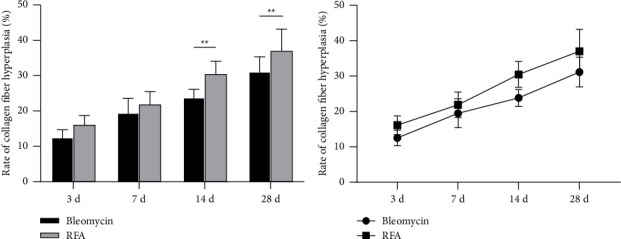
(a) Comparison of the rate of collagen fiber hyperplasia between the bleomycin group and radiofrequency ablation (RFA) group on Days 3, 7, 14, and 28 after treatment; (b) trends of collagen fiber changes on Days 3, 7, 14, and 28 after treatment. ^∗∗^Compared with the bleomycin group, *p* < 0.01.

The collagen fiber hyperplasia in the comb was examined at the 3rd, 7th, 14th, and 28th days posttreatment. Both groups showed a gradual increasing trend in collagen fiber hyperplasia rates over time (Table 2, Figure 9). Pairwise comparisons revealed no significant difference (*p* > 0.05) in collagen fiber hyperplasia rates between the bleomycin group and the RFA group at 3 and 7 days posttreatment. However, significant differences (*p* < 0.01) were observed between the bleomycin group and RFA group at 14 and 28 days posttreatment. The results indicated that, in the long term, the collagen fiber hyperplasia was more pronounced in the RFA group compared to the drug injection group.

## 4. Discussion

In vascular abnormalities, skin hemangioma and vascular malformations are the most common diseases [1–3]. Although the classification system, recommendations, and guidelines have been established and continuously updated, there are still problems in diagnosis and treatment [4]. There are many clinical treatment methods, such as drug therapy and physical intervention, but it is still necessary to continuously explore effective and safe treatment methods. Comparing the effectiveness of different treatment methods helps to provide a better strategy for the treatment of vascular lesions [5–7]. Dalili proposed that RFA can increase the intracellular temperature to more than 50°–55° C, resulting in irreversible cell death, complete closure of small blood vessels, reduction of blood vessel diameter, and partial blood flow limitation of larger blood vessels [20]. This is a very promising vascular closure treatment. In a limited number of cases, RFA has shown promising results in the treatment of vascular abnormalities [21, 22], but its potential pathophysiological effects in animal models have been rarely studied.

In this study, the cockscombs of the RFA group were equipped with a unipolar device (XL-RFA device; Beijing, China, Wang Xinglin) for treatment, the control group of cockscomb injection of bleomycin (with 1 mg/mL concentration bleomycin administered per site). The specific operation method has been reflected in the text under “Materials and Methods”. Uniform operating parameters and methods were selected, and the untreated side of the comb was used as a self-control, which effectively solved the problem of experimental error caused by the difference between the comb itself and different roosters. It is reported that the effective rate of bleomycin is 43%–82.7% [23–25], which is the gold standard treatment for some vascular abnormalities. In this study, we used bleomycin treatment as a control to study the efficacy and related pathological mechanisms of RFA. A self-developed single-electrode RFA device was used in the experiment. The size of the ablation area depends on several important factors, including generator power, electrode selection, and treatment location and time. When the treatment temperature reaches > 60°C, coagulation necrosis and cell death will occur in venous malformation tissues, resulting in vascular and neurological diseases and blood coagulation. In terms of model selection, the cockscomb is a mature model because its subepidermal vascular system is similar to human skin hemangiomas and vascular malformations in terms of morphology, structure, and abundant capillary networks [17, 18]. In this study, we used vascular endothelial cells in the proliferative state of developing roosters, and therefore, can represent common skin hemangiomas and vascular malformations.

In this study, the bleomycin injection group showed significant tissue edema, while the RFA group significantly reduced these side effects. On the 3rd, 7th, and 14th days, the edema in the RFA group was significantly less than that in the bleomycin group, which can be clearly seen from the gross observation. Although there was no significant difference in edema between the two groups at 28 days, the reduction of side effects during the treatment was of great significance for clinical application. Since bleomycin is still the first-line therapy for the treatment of abnormal vascular lesions, the reduction of side effects can reflect the clinical benefits of RFA under the premise that the two can produce the same effect. This finding provides an important basis for clinical selection of appropriate treatment methods [12, 26]. Future studies can also further explore the combined effects of drug therapy and physical intervention and optimize drug dose and radiofrequency treatment parameters in order to improve the overall effect of the treatment.

In this study, it was found that the scab formation in the RFA group was earlier and the scab boundary was clearer than that in the bleomycin group. At a later time point, most of the crusts in the treatment area of the RFA group fell off, and the chicken crown tissue after treatment showed a grayish-white circular area with clear boundaries. In contrast, although most of the cockscomb crusts in the bleomycin group fell off, the tissue boundary was not clear. This part of the discovery shows that for some hemangiomas and vascular malformations with high cosmetic requirements, RFA can accurately remove the lesions [27] and can show a good cosmetic effect, which is also an important advantage of RFA in clinical treatment.

In the study of bleomycin injection in the treatment of vascular lesions, the number of capillaries was observed to decrease gradually, while in the RFA group, it showed a different trend. It should be noted that at 3 days after treatment, the capillaries in the RFA group were significantly reduced (reduction rate of 71.4%), while in the bleomycin group, the reduction rate was 33.5%. In the following time, the number of blood vessels in the RFA group gradually increased, and the number of blood vessels in the bleomycin group gradually decreased. Although there was no significant difference in the capillary rate between the two groups at 28 days, the number of blood vessels in the RFA group was always smaller than that in the bleomycin group at 3, 7, and 14 days. The above results show that bleomycin and RFA affect the vascular structure of the comb in different mechanisms. The former shows a gradual process of destroying blood vessels for blood vessels, while the latter destroys capillaries in a short time through physical factors, and finally achieves the same therapeutic effect of capillary reduction. This finding provides an important basis for understanding the pathological changes of blood vessels and suggests the clinical application value of different treatment methods. The follow-up study can further explore the mechanism of bleomycin and RFA on vascular endothelial cells, so as to provide more clinical treatment.

In terms of collagen fiber proliferation, the collagen proliferation rate of the RFA group was significantly higher than that of the bleomycin group at 14 and 28 days, which were 23.93% and 31.28% of the bleomycin group and 30.62% and 37.20% of the RFA group, respectively (*p* < 0.01). This result indicates that RFA may promote the biosynthesis of collagen fibers and replace the vascular components of hemangioma by collagen fiber hyperplasia, which provides a new idea for improving tissue healing and the treatment of vascular lesions. Future research can further explore the relationship between collagen proliferation and tissue healing, as well as the dynamic changes of collagen fibers at different time points. Through these studies, it is expected to further optimize the treatment of vascular related diseases and improve the clinical effect.

The limitations of this study are mainly reflected in the relative small sample size, which may limit the universality of the results. In addition, although we have evaluated the short-term effects of different treatments, there is a lack of in-depth study of their long-term effects, which may affect our understanding of the comprehensiveness of the results. Further studies need to expand the sample size and include long-term follow-up data to more comprehensively evaluate the effects of bleomycin injection and RFA in the treatment of cockscomb vascular lesions.

## 5. Conclusions

In this study, RFA and bleomycin treatment were compared to reveal the different mechanisms of the two treatments in terms of pathophysiology and histopathology. It is confirmed that RFA has the advantages of precise treatment, controllable depth, and small side effects. These findings not only enrich the understanding of the treatment of vascular diseases but also provide an important reference for optimizing the treatment strategies of vascular-related diseases in the future. Considering the therapeutic effect and side effects, RFA shows better clinical application potential. RFA and RFA combined with drugs in the treatment of hemangioma and venous malformation may be an ideal method in clinical and basic research, which needs further study.

## Figures and Tables

**Table 1:** Comparison of the rate of capillary reduction (%) between the bleomycin group and radiofrequency ablation (RFA) group at different time points (mean ± standard deviation, *n* = 8).

Groups	3 d	7 d	14 d	28 d
Bleomycin	33.49 ± 3.55	40.07 ± 3.38	49.79 ± 5.13	58.60 ± 5.63
RFA	71.41 ± 4.56^∗∗^	67.14 ± 4.15^∗∗^	62.65 ± 4.16^∗∗^	60.69 ± 5.24
*F*	0.878	0.719	1.317	0.025
*p*	< 0.01	< 0.01	< 0.01	> 0.05

^∗∗^Compared with the bleomycin group *p* < 0.01.

**Table 2:** Comparison of the rate of collagen fiber hyperplasia (%) between the bleomycin group and radiofrequency ablation (RFA) group at different time points (mean ± standard deviation, *n* = 8).

Groups	3 d	7 d	14 d	28 d
Bleomycin	12.54 ± 2.23	19.58 ± 4.11	23.93 ± 2.39	31.28 ± 4.22
RFA	18.87 ± 11.43	24.35 ± 10.34	30.62 ± 3.65^∗∗^	37.20 ± 6.21^∗∗^
*F*	1.342	0.625	4.201	4.897
*p*	> 0.05	> 0.05	< 0.01	< 0.01

^∗∗^Compared with the bleomycin group, *p* < 0.01.

## Data Availability

All data generated or analyzed during this study are included in this published article.
